# Recent Advances in Fiber–Hydrogel Composites for Wound Healing and Drug Delivery Systems

**DOI:** 10.3390/antibiotics10030248

**Published:** 2021-03-02

**Authors:** Marta O. Teixeira, Joana C. Antunes, Helena P. Felgueiras

**Affiliations:** Centre for Textile Science and Technology (2C2T), Department of Textile Engineering, University of Minho, Campus of Azurém, 4800-058 Guimarães, Portugal; martasofia.teixeira@hotmail.com (M.O.T.); joana.antunes@2c2t.uminho.pt (J.C.A.)

**Keywords:** fiber–hydrogel composite, biodegradable polymers, skin regeneration, drug delivery platforms, controlled release

## Abstract

In the last decades, much research has been done to fasten wound healing and target-direct drug delivery. Hydrogel-based scaffolds have been a recurrent solution in both cases, with some reaching already the market, even though their mechanical stability remains a challenge. To overcome this limitation, reinforcement of hydrogels with fibers has been explored. The structural resemblance of fiber–hydrogel composites to natural tissues has been a driving force for the optimization and exploration of these systems in biomedicine. Indeed, the combination of hydrogel-forming techniques and fiber spinning approaches has been crucial in the development of scaffolding systems with improved mechanical strength and medicinal properties. In this review, a comprehensive overview of the recently developed fiber–hydrogel composite strategies for wound healing and drug delivery is provided. The methodologies employed in fiber and hydrogel formation are also highlighted, together with the most compatible polymer combinations, as well as drug incorporation approaches creating stimuli-sensitive and triggered drug release towards an enhanced host response.

## 1. Introduction

Biomaterials are defined as nonviable materials, with potential for applications in medical devices, that possess the ability to interact with biological systems to evaluate, treat, replace or enhance the performance of any tissue [1]. Biomaterials are classified in different ways; the most common refers to their chemical nature and is subdivided in metallic materials (ferrous and non244-ferrous) and non-metallic materials (organic: polymers, biological materials, and carbons; and inorganic: ceramics and glasses). Composites are considered another very important class of biomaterials and result from the combination of two classes of materials that work in synergy to improve the properties of the final product above those of the individual components [1,2].

The continued research in this field has raised the specificity level of the biomaterials developed and, therefore, has increased its impact in the healthcare global market [1]. Polymers represent a large portion of all biomaterials used in the biomedical field (about 45%) [2], and their application appears to have no end. They can be processed in the form of particles, foams, films, membranes, hydrogels and fibers, and combinations of these 3D structures can then be made to generated intricate, target-direct, specialized biomedical systems. Biomedicine has resorted to these constructs to understand specific biological processes and to engineer high-performance therapies to treat a variety of diseases. The need to match the desired functions/characteristics of a given tissue or cell has driven the combination of different classes of biomaterials in complex constructs (e.g., fiber–hydrogel composite) that can effectively respond to the local demands and provide the necessary tools to reach the desired goals. In recent years, fiber–hydrogel composites have been disclosed as one of those systems that combine different structures to improve individual features and enhance inherent advantages to achieve successful outcomes. In biomedical engineering, the importance of these constructs is particularly noticeable in wound healing and drug delivery. In both areas, fiber–hydrogel composites can be a good alternative to the use of antibiotics and/or their controlled administration.

The present review explores this subject further, starting with the introduction of basic concepts associated with polymer properties and processing in the form of fibers and hydrogels and then evolving towards the combination of these two structures in one to successfully respond to specific needs. The most recent studies highlighting fiber–hydrogel composites are here identified, giving particular attention to the engineering of wound dressings and drug delivery systems.

## 2. Polymers Natural/Synthetic

The word polymer is derived from the Greek *poly* and *meros*, meaning many and parts, respectively. Polymers are macromolecules that result from the repetition of smaller molecules, the monomers [3]. The nature of the monomers and the specific bonds generated between them, and their spatial rearrangement, determine the properties of the built polymer [4]. The process through which a polymer is formed is named polymerization and can be described as a chemical reaction in which the combination of one or more monomers occurs [3]. Polymers can also be biologically derived or synthetically produced [2]. Natural polymers are created in nature during the life cycles of biological systems, such as plants, microorganisms, and animals [5]. These polymers are widely used in scientific community, namely, in tissue engineering, wound dressing and drug delivery systems [6,7,8], due to their biocompatibility, non-toxicity, biodegradability and bioactivity, particularly their inherent anti-inflammatory and antibacterial properties [9]. These polymers include polysaccharides and polypeptides. Polysaccharides, the most abundant class of biopolymers, are polymeric carbohydrate molecules formed by glycosidic bonds with different structures and properties depending on molecular weight and chemical composition [8]. In particular, polysaccharides, compared to polypeptides, are generally more stable and usually do not denature on heating [10]. Regarding their chemical properties, they have polyfunctionality, high chemical reactivity, chirality, chelation and adsorption capacity, which allow them to be chemically and biochemically modified very easily. These modifications result in different polysaccharide derivatives, which increase the range of applications [6,8,11]. The alginate, hyaluronic acid (HA), cellulose and chitosan (CS) stand out between the polysaccharides for being the most used in biomedicine (Table 1). Just like polysaccharides, polypeptides are produced by microorganisms. Polypeptides are macromolecules composed of repeated units of amino acids linked by peptide bonds. Their versatility, flexibility, good performance in metabolic adaptation and imitation of the extracellular matrix makes them good candidates for tissue scaffolding and drug/gene delivery [12]. The most common polypeptides used in biomedicine are collagen and gelatin (Table 1). However, known limitations of natural polymers include their very low dimensional stability, susceptibility to immunogenic responses, possibility of pathogen transmission and high batch-to-batch variability [12,13]. For this reason, biodegradable synthetic polymers are frequently employed as alternatives. 

Indeed, some of the key benefits of synthetic polymers are their reproducibility, which allows mass production, and their ability to be tuned according to specific requirements. Their degradation profile can also be easily manipulated via their hydrolytic groups [14], even though bulk degradation can occur [15]. Moreover, synthetic polymers are biologically inert, thus without a therapeutical impact, but may induce chronic inflammation [16]. In biomedicine, poly(ethylene oxide) (PEO), poly(ε-caprolactone) (PCL), polylactic acid (PLA), poly(lactic-co-glycolic acid) (PLGA), poly(vinylpyrrolidone)(PVP) and poly(vinyl alcohol) (PVA) [15,17] constitute the most studied polymers in the field (Table 1) [18]. They may additionally be combined with natural polymers. Hybrid polymers can result from the total or part combination of natural and synthetic polymers. As is the case of the combination of the PLGA (synthetic polymer) and the CS (natural polymer) that result in the PLGA-CS hybrid polymer that has been studied in several areas, namely in therapeutic delivery [19]. The choice of polymers for the formation of scaffolds, based on their characteristics, has proved to be crucial in the properties and applicability of the final scaffold. Currently, synergisms between synthetic and natural biomaterials in the form of 3D scaffolds, such as hydrogels and nanofibrous mats, are in high demand for biomedical applications, being frequently preferred over constructs made of polymers belonging to only one of these categories [7,15,20].

## 3. Hydrogel

Hydrogels are 3D networks of hydrophilic polymers capable of absorbing and retaining significant amounts of fluids [58], which have also been widely applied in wound healing [30,59]; cartilage tissue engineering [36,60]; bone tissue engineering [61]; and delivery of proteins, growth factors and antibiotics [20,62]. 

Hydrogels can be classified based on their source, namely the composing polymers, in natural or synthetic (Table 2). Thus, nature-derived hydrogels may consist of natural polysaccharides or polypeptides [6,12], ergo carrying molecular recognition sites enabling cell/tissue communication pathways and modulation towards a therapeutical effect [63]. However, as hydrogels, they tend to present low stability in aqueous medium, poor mechanical properties and quick degradation rates [63]. On the other hand, hydrogels based on synthetic polymers are typically mechanically resilient and display superior elastic properties. Still, their biological inertness, blocking any chances of tuning cell behavior towards a healthier state, limits their use in biomedicine [63,64]. Hybrid hydrogels, combining natural and synthetic polymers [65], have been proven useful to create smart hydrogels (alginate-g-(PEO-poly(propylene oxide)-PEO) [66]), in biomedical materials (PVA/collagen [67]) and in tissue engineering applications (CS/PCL [47]), to name a few examples. Their polymer composition may also subdivide hydrogels in homopolymers, copolymers, multipolymers or interpenetrating polymer networks (IPN) [68]. Homopolymer hydrogels are made of crosslinked polymer networks derived from a single type of basic structural unit (monomers) [69]. Copolymer hydrogels are frequently crosslinked polymer networks made up of two co-monomer units with at least one hydrophilic component (not soluble in water). These networks can assume three types of configuration, arbitrary, block or may alternate between both along the chain [70,71]. Multipolymer hydrogels are the result of the reaction of three or more co-monomers [72]. In turn, IPNs are an important class made of two independent crosslinked synthetic and/or natural polymer components, in which a new hydrogel polymeric network is polymerized within a pre-existent [68,73]. In case only one polymer network from the two is crosslinked, the hydrogels are designated as semi-IPNs. [68]. 

Hydrogels may also be categorized as amorphous, crystalline or semi-crystalline, depending on their physical organization and chemical composition. Semicrystalline hydrogel networks are mixtures of crystalline as well as amorphous phases [74]. These properties may also affect the hydrogel degradation rate, sub-divided in degradable or non-degradable structures [68]. Most hydrogels used in tissue engineering and drug delivery systems are biodegradable and are developed to degrade into biologically acceptable molecules (non-toxic degradation biproducts) [75,76]. The degradation rate of biodegradable hydrogels may be manipulated via the polymers’ molecular weight [77], by the action of oxidizing agents [78], or by the presence of enzymes [79]. Tanan et al. developed a semi-interpenetrating hydrogel (semi-IPN) consisting of a mixture of cassava starch-g-polyacrylic acid/natural rubber/PVA. This hydrogel exhibited an excellent water retention capacity and proved to be highly sensitive to salt concentration, type of cations, pH and swelling time. In addition, it demonstrated good biodegradation with a rate of 0.626 wt.%/day [80].

In terms of their physical properties, hydrogels can be categorized as conventional or smart. Conventional hydrogels are characterized by low response rates, in general. They have a very low swelling rate due to their small matrix size. This limitation has triggered a greater interest in macroscopic hydrogels, where the size of the pores allows a higher swelling rate. Smart hydrogels are hydrogels that react to changes in environmental conditions (external stimuli) by swelling or reversibly collapsing [81,82]. Hydrogels can be physical, chemical or biochemical/biological in relation to the type of response/stimulus [68]. Physical stimuli like temperature, electric field, magnetic field, light and pressure and chemical stimuli like pH, solvent composition and ionic strength can change the swelling state of the hydrogel. Hydrogels with biochemical/biological responses are capable of interacting with the surrounding environment [81,83]. In terms of production, hydrogels can be formed by physical [30] and/or chemical [21] crosslinking of polymers, which will be discussed in the following sections. Hydrogels can also be classified based on their charge in non-ionic (neutral), ionic (anionic or cationic), amphoteric (acidic and basic groups) or zwitterionic (anionic and cationic groups in each structural unit) [68].

Hydrogels benefit from a high degree of flexibility, adjustable viscoelasticity, biocompatibility, high permeability to oxygen and essential nutrients, high water content and low interfacial tension with aqueous medium [7,22]. The hydrogel biocompatibility, that is, its ability to perform its intended function without inducing side effects in the host, is one of its most crucial characteristics. Further, in case of wounds, for instance, their limited adhesion may allow removal from the wound bed without causing additional trauma or destroying the newly formed tissues [84,85].

Certain hydrogels even have capacity to alter their swelling state in response to environmental variations; these function as triggers to change the physical and/or chemical properties of the hydrogel. For example, in the case of pH-sensitive hydrogels, the polymers that make up the hydrogel contain hydrophobic moieties that swell in water according to the pH of the external environment. Thus, in the absence of this stimulus, the hydrogel maintains its initial swelling state [81]. This property makes them good candidates for drug delivery systems. In this case, altering the swelling state in response to a change in pH opens opportunities for controlling the timing of drug release. Kwon et al. described the synthesis via chemical crosslinking of pH-sensitive hydrogels based on hydroxyethyl cellulose and HA for transdermal delivery of the drug isoliquiritigenin. At pH 7, the electrostatic repulsions between the carboxylate groups of HA lead to the enlargement of mesh and, consequently, to an increase in the amount of isoliquiritigenin released. The authors observed an efficacy greater than 70% of the release of the drug due to the pH and excellent adhesive properties of the hydrogel, which makes it a good candidate for treating skin lesions [86].

### Hydrogel Formation: Techniques

Considering that many hydrogels degrade very easily in biological systems or in contact with water-based fluids, the purpose of the crosslinking process is to improve the insolubility, mechanical strength, and rigidity of the polymer network. Hydrogels can be physically or chemically crosslinked (Table 3) [87]. Physical hydrogels are networks with transient junctions (reversible connections), traditionally disordered and fragile. They result from interactions such as ionic bonding [88], hydrogen bonding [89], hydrophobic interactions [90], and crystallization [91]. The physical properties of the polymers and the gelation conditions determine the internal structure of the hydrogel, by modulating properties such as gel density, porosity and mechanical performance (e.g., rigidity) [92]. Physical hydrogels tend to exhibit low mechanical strength and are often unstable [93]. The dissolution of physically crosslinked hydrogels can occur in response to changes in temperature, application of stress, ionic strength, pH and solvent composition. Because of their reversible character, the polymer solution resulting from the dissolution process may undergo again gelation and restore the original hydrogel features [65,94]. 

Unlike physical, chemical hydrogels are polymer networks with permanent junctions, formed via covalent bonds, which are capable of maintaining the structure integrity for longer (increased degradation time) [95]. Chemically crosslinked hydrogels are known to be mechanically strong. However, although they present a permanently fixed shape, they have low fracture resistance and extensibility [93]. Further, certain chemical crosslinking agents are toxic and can cause adverse reactions; thus, they must be extracted from the gels before use [96]. Photopolymerization, enzymatic crosslinking, crosslinking molecules and polymer–polymer crosslinking are the four major chemical crosslinking methods that can be employed to form crosslinked hydrogels. 

Hybrid hydrogels result from the combination of physical and chemical crosslinking of polymers. These double crosslinked hydrogels combine the advantages of both strategies, namely, low surface tension, remarkable thermodynamic stability and elevated capacity of solubilization [65,93]. 

Various chemical and physical hydrogels have been prepared from natural and/or synthetic polymers for a variety of biomedical purposes. Chitosan hydrogels formed with the crosslinking agent trisodium salt 6-phosphogluconic (6-PG-Na^+^) loaded with the drug piroxicam were developed by Martinez-Martinez et al. The interaction between ionic polymer cationic groups and anionic groups of the 6-PG-Na^+^ crosslinker led to the formation of ionic hydrogels. The authors observed that the hydrogel had potential as a drug vehicle for topical administration since at pH close to neutrality there was less degradation than at lower pH, with a release of 90% of piroxicam during 7 h (release controlled by pH). This hydrogel proved to be a good candidate as a wound dressing given its good adhesion properties, non-toxicity and ability to induce healing and regeneration [115]. In another study, Wang et al. developed a hydrogel based on gelatin methacrylamine/poly(ethylene glycol)diacrylate (GelMA/PEGDA) via photo-crosslinking (with photoinitiator I2959). The engineered hydrogel was shown to have stronger mechanical properties than pure GelMA hydrogels and a degradation rate that lasted 4 weeks. Here, osteoblasts were able to adhere and proliferate along the surface, showing great cell viability and biocompatibility. Such characteristics make this hydrogel a good candidate for guided bone regeneration [116]. Table 4 lists some of the most recent examples physical, chemical and hybrid hydrogels employed in biomedicine and their respective production techniques.

## 4. Fiber

The use and production of polymer-based fibers by humans has been described since pre-historic times. The earliest account of the biomedical use of fibers is suggested in decorations of the Tassili caves, engraved between 5000–2500 BC [131]. Ancient records, date the beginning of the use of cotton to the first half of the 6th millennium BC and the cultivation of silkworms to produce silk fibers to the 4th millennium BC [132,133]. With the industrial revolution, there was a need to create more efficient fiber production strategies. In the 14th century, the spindle to manufacture wool and cotton fibers emerged. The evolution in this field did not stagnate and the production of fibers continue evolving until the 19th century, dramatically increasing the use of natural fibers in the 1940s [11,134]. Years later, in the middle of the 20th century, the production of synthetic fibers began [11]. Nowadays, this area is constantly evolving, being already available several high precision methods of fiber production [133,135]. The application of fibers in biomedicine occurs in several areas, namely in wound dressings [136], bone tissue engineering [137], drug-controlled release [138], among others.

Fibers can be divided in two classes, natural and synthetic. Natural fibers can be extracted from plants, animals or minerals. Synthetic or man-made fibers usually arise from chemical processing [135]. In general, all plant-derived fibers are composed of cellulose, while animal-derived fibers contain proteins [139]. Natural fibers are made of millions of macrofibrils, which in turn are formed by microfibrils [140], composed mainly of crystalline cellulose (30–90%, that varies depending on the part of the plant concerned) surrounded by an amorphous matrix of lignin and hemicellulose [141]. These three fiber components are linked together by covalent bonds [140], with the fiber properties being defined by their composition, microfibril angle, crystallinity and internal structure. The stiffness of the fibers depends essentially on the angle of the cellulose microfibrils, the smaller the angle the greater the stiffness. Other properties, such as water absorption, moisture resistance, swelling and integration of the fiber bundle are determined by the other components, like hemicellulose [141]. In general, vegetable fibers are characterized by their biodegradable nature, lightweight, renewable capacity, abundance, improved mechanical properties, low cost and low density [142,143]. Because of these characteristics, natural fibers can be processed in various forms, including rope, yarn and reinforcing agents for biocomposites [144]. However, as reinforcements, the quality and efficiency of the final product are dependent on environment conditions which may be unpredictable from batch to batch, generating heterogeneity between fibers with the same origin [142]. Cellulose nanofibers have been applied in areas such as drug delivery [145] and tissue engineering [146]. Doench et al. reported the development of non-cellularized injectable suspensions of viscous CS solutions, filled with cellulose nanofibers as a strategy for visco-supplementation of the intervertebral disc nucleus pulposus tissue [146]. Natural fibers derived from animal sources can be collected from wool, silk and hair, for instance [139]. In the case of wool, depending on the animal it is collected from, be it sheep, lama or rabbit, there are properties that vary, namely, the color and the weight of the fibers [147]. Keratin is the main component of wool and hair [148]. This protein has excellent biocompatibility, biodegradability and is capable of increasing scaffolds elasticity and mechanical resilience by self-assembly and polymerization [149]. Silk fibers, on the other hand, are mainly made up of two structural proteins, fibroin (mechanical strength) and sericin (coating) that can be organized in a linear structure [150]. Silk fibers are characterized by being biodegradable and biocompatible. Although in the past their use was limited to clothing, today, silk is used in surgical knits, sutures, and wound healing. In addition, several researches are now in course to examine their use in films, scaffolds, electroplated materials and hydrogels [133,151]. In fact, the increase in research on polymeric composites reinforced with natural fibers has emerged side by side with the use of synthetic fibers in polymeric composites [143]. 

Synthetic fibers can be classified in inorganic or organic. Inorganic fibers are those that are not made of organic compounds [152]. As such, organic fibers can be manufactured either from natural or synthetic polymers. Most of the fibers used are of polymeric origin. Thus, the molecular weight of the polymer fiber plays a crucial role in influencing the tensile strength and the physical properties of the final construct [153]. Synthetic polymer fibers can be prepared from various polymers, as can be seen in Table 5 [153]. However, in biomedicine, those endowed with biodegradable features attract much more attention, namely, the PLA and the PCL polyesters [154]. PLA has the potential to replace fossil-based polymers [139]. It is biocompatible and its degradation biproducts are non-toxic, which favors its application in health-related fields [155]. On its turn, PCL is a biocompatible, linear polyester with improved elastic properties (despite having low tensile strength, it is capable of very high elongation) [154] that make it highly desirable for tissue engineering systems [156]. 

Numerous researches describe the combination of synthetic polymers and natural polymers as the key for a successful fiber production [157,158,159]. For instance, Hu et al., reported the production of alginate/PCL composite nanofibers by co-electrospinning to enrich cancer stem cells (CSCs) constructs. The author studied the impact of the separated PCL and alginate fibers and the alginate/PCL composite having observed that the application of composite fibers is more effective in selecting cells than pure fibers. The fact that these scaffolds can be adjusted (composition proportion) to isolate CSCs from different tissues may potentially facilitate cancer research [157]. Levengood et al. developed CS/PCL nanofiber structures that combined the biological properties of CS and the stability and mechanical integrity of PCL for prospective applications in skin tissue engineering. Throughout the study, it was found that the nanofiber structure increased the wound healing rate, promoted general closure, re-epithelialization, maturity of the neoepidermis and collagen deposition when compared to the control. Such facts strengthen the potential of CS/PCL nanofiber structures for skin repair [158]. The other section of synthetic fibers, the inorganic fibers, can be subdivided in three main groups, which are the metals and alloys, the metal or semi-metal compounds and the carbon-based fibers (Table 5). 

Many of the inorganic fibers generally exhibit high strength, high thermal and chemical stability and stability against any kind of organic solvent [152]. Regarding fiber glass, they have a relatively low cost, high tensile strength, high chemical resistance and good insulation properties. In case of carbon fibers, these have numerous advantages, such as high stiffness and tensile strength, high chemical resistance, high temperature tolerance, present low cost and low thermal expansion. Because of these characteristics, both glass fibers and carbon fibers are often used as reinforcement in polymeric composites [143]. These fibers can be combined with other components. Naskar et al. described a composite of regenerated silk protein fibroin reinforced with functionalized carbon nanofibers, loaded with growth factors (BMP-2 and TGF-β1) essential to bone regeneration. The matrices formed were porous, immune-compatible and bioactive when incubated in simulated body fluid. Here, it was seen that the reinforcement of the nanofibers influenced the mechanical property of the matrices, increasing the compression module up to 46.54 MPa [160].

Fibers can be classified according to their internal structure (uniform fibers or core-shell) or orientation (aligned or arranged randomly). They can also be formed of continuous monofilament yarns or multifilament yarns. Both natural and synthetic fibers can be characterized physically (diameter, length, density and moisture gain) and mechanically (tensile strength, specific strength young’s modulus, specific young’s modulus and failure strain) [161]. Natural fibers have moderate mechanical properties, high thermal sensitivity, low density, acceptable modulus-weight ratio, low cost, can be extracted from unlimited sources, and display good recyclability and biodegradability. However, the high sensitivity to humidity, higher variability of physical and mechanical properties and low durability are some of the disadvantages of natural fibers. In turn, synthetic fibers have high mechanical properties, low sensitivity to moisture and low thermal sensitivity. Limited sources and moderate recyclability are some disadvantages of synthetic fibers. [162,163]. Even though their mechanical resilience is highly attractive, the energy necessary to produce synthetic fibers tends to be more than that required for natural [140]. 

### Fiber Production: Techniques 

As explained earlier, the fiber final properties depend on the polymer composition. However, they are also dependent on the processing conditions. The four most used fiber production methods include electrospinning, melt-spinning, wet-spinning and dry-spinning (Table 6). The electrospinning is a technique that allows the generation of polymeric fibers with submicron or nanometric diameters while conventional techniques such as melt-spinning, wet-spinning and dry-spinning can produce polymer fibers with diameters up to the micrometer range.

Depending on the fiber production method employed, several precautions must be taken into consideration, such is the case with the processing of CS fibers via wet-spinning. CS fibers have a very low tensile strength (due to their increased hydrophilicity) and, therefore, chemical crosslinking must be induced by Epichlorohydrin (ECH) to improve its wet tenacity [178]. According to the chemical (e.g., composition and rate of degradation) and physical (e.g., diameter, strength and porosity) characteristics, the electrospun nanofibers can guide and interact with the injured tissue to improve wound healing [179]. 

## 5. Fiber–Hydrogel Composites 

As seen in the previous sections, both hydrogels and fibers display great potential in biomedicine, particularly in the wound healing and drug delivery areas [88,156,157,180]. Despite the many advantages that make these scaffolding systems promising, there are still aspects that often limit their application. For instance, the low mechanical stability of natural hydrogels and the not-so-great biocompatibility of synthetic hydrogels tend to constrain their uses [63]. In the case of fibers, there is a limitation associated with the lack of 3D network formations which can restrict cell migration/infiltration [181]. Given these limitations, a number of researches are now dedicated in combining the advantages of fibers and hydrogels to produce an optimal, highly functional composite system [182,183,184]. In this sense, the objective of these investigations is to optimize the mechanical/biological functionalities of composites by promoting the combination of beneficial properties of both components (fiber/hydrogel) and reducing the impact of their undesirable features in the final application. The mechanical properties of hydrogels, in this case fiber–hydrogel composites, are significantly influenced by the addition of fibers [185], as they serve as a structural support for the hydrogel to surround, for instance [184]. Regev et al. reported that the incorporation of bovine serum albumin fibers in dextran/gelatin hydrogels increases the elasticity modulus of the hydrogel and decreases its gelation time [186]. Gelatin nanofibers aligned and infiltrated in alginate hydrogels may also increase the tensile modulus and rigidity of the overall hydrogel construct [187].

**Figure 1:**
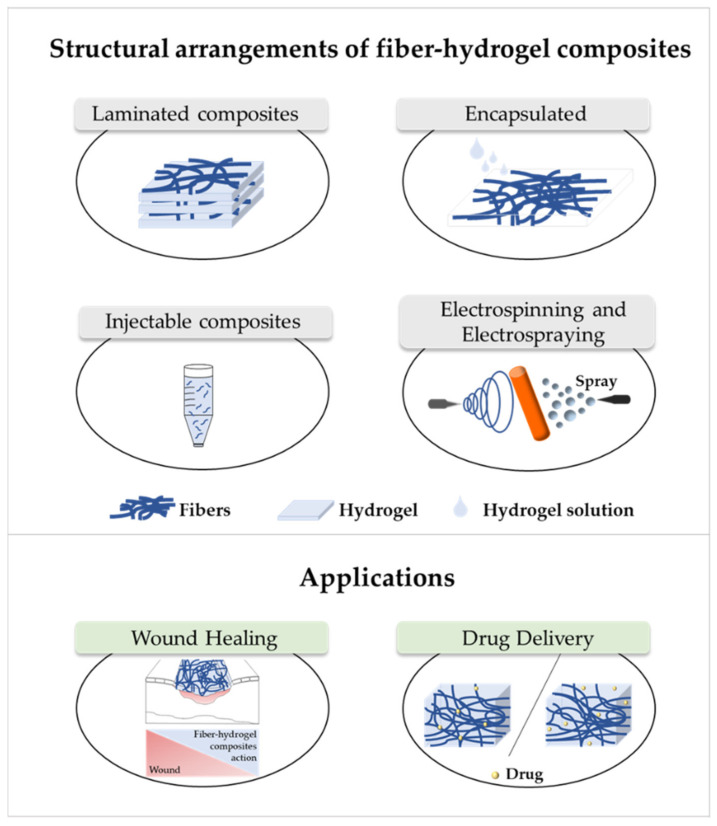
Structural arrangements of fiber–hydrogel composites and their applications in wound healing and drug delivery. Fiber–hydrogel composites with laminated structure result from the junction of individually manufactured fibers and hydrogels that can be organized in layers with different orientations. The encapsulation of fibers in hydrogels can result from crosslinking of the hydrogel solution directly into the fibers. In case of injectable composites, small individual fiber fragments are added to the hydrogel solution, resulting in an encapsulated and injectable composite structure. These composites can also be formed by the simultaneous combination of electrospinning and electrospraying applied directly towards a single collecting system (shown in orange).

The fibers used in fiber–hydrogel composite can have different origins, natural or synthetic, and, at a morphological level, they can also differ depending on the desired application. Generally, the fibers used in these composites can be classified as long or short, and within the composite, they can exhibit a continuous or discontinuous pattern. Specifically, long and continuous fibers produced by electrospinning tend to possess small pores that limit cellular penetration and growth [188]. Based on the potential application of the scaffold, the organization of the fibers is a crucial element for the performance of the intended function, which can be oriented uniformly or randomly [185]. Although the available literature is still limited, several methods of combining fibers with hydrogels for the creation of composites with different structures have been reported. Of all, the most common arrangements of composite fiber–hydrogel structures are the stacked, with hydrogels and fibers forming layers (laminated composites) [189], the encapsulated, with fibers being enclosed within the hydrogel matrix [190], the injectable composites [191] and the electrospinning and electrospraying combination [192] (Figure 1). In fiber production, electrospinning is one the most used techniques due to its simplicity, cost efficiency, flexibility, scalability the advantage of mimicking the natural extracellular matrix (ECM) [16,166,193,194], so its combination with hydrogel fabrication methodologies is very frequent. 

Laminating is the simplest method to yield a fiber–hydrogel composite scaffold. Laminated composites consist of the junction of individually manufactured hydrogels and fibers in different layers. The number of fiber layers influences the mechanical properties of the composite. These composites can be formed by a single layer of fibers or by multilayers, with different orientations (e.g., 0°, 45° and 90°). The orientation of the fibers within the composite allows to control the toughness and strength of the final structure. These constructs exhibit significantly improved tensile properties compared to hydrogels alone [195]. However, they can undergo delamination very easily after water absorption due to the weak interactions between layers [196]. Additionally, the 2D structure of the fibers becomes a limitation for applications where it is essential to mimic the ECM, since this structure makes cell migration very challenging [197]. Encapsulating fibers in hydrogels can be accomplished by crosslinking the hydrogel directly into fibers with a pre-determined architecture or by immersing the fibers in a hydrogel precursor solution. In this process, the gaps between the fibers are occupied by the hydrogel precursor solution that later crosslinks. Based on encapsulation fibers in hydrogel, McMahon et al. hypothesized a composite with a tubular structure with circumferential mechanical properties similar to coronary artery vessels [187,190]. Papaparaskeva et al. projected prefabricated fibrous mats of PVP/silver nanocomposites incorporated within semi-IPN hydrogels in two unique forms of laminated dispersion (a prefabricated electrospun fibrous mat was placed in the circumference of the fiber/hydrogel composite) and homogeneous (a 2D circular fibrous mat was homogeneously encapsulated within a 3D hydrogel matrix). They noted that the dispersion mode of electrically spun fibrous mats within the hydrogel significantly influences the mechanical performance of the resulting composite [198]. Injectable composites have been considered an alternative to produce fiber–hydrogel constructs with homogeneous qualities. In this type of composite, small individual fiber fragments (smaller sizes facilitate injectability) are added to a hydrogel precursor solution. Subsequently, these fibers are incorporated into the crosslinked hydrogel matrix (in the desired environment), playing a reinforcing role. This production strategy is minimally invasive; however, the absence of connections between fibers can become a restriction for certain applications [188,199]. The electrospraying process has been used to form fiber–hydrogel composites. Here, the hydrogel solution is sprayed in fine droplets on fibers produced by electrospinning. These drops, which are deposited on the fibers, can have different sizes, from nanometers to several micrometers. The electrospinning–electrospraying has therefore low cost and is easy to operate. Furthermore, it allows to obtain a composite fiber–hydrogel with different structures and with adjustable size and morphology [200,201,202]. Despite the formation processes of fiber–hydrogel composites mentioned above, which are already used in investigations, a less positive aspect can be highlighted. This focuses on the differences in hydrophilicity between the fibers and the hydrogels that can cause some incompatibility, which then may result in a separation of the compound. In this sense, the modification of the fiber surface is considered a potential solution to improve this limitation [203,204,205].

There are several approaches that have been used to improve the properties of fibrous scaffolds and hydrogels, namely the development of fiber–hydrogels composites (Figure 1). As seen, these composites continue to conform to the same guidelines applied for the fabrication of the individual parts, with peculiarities related to their fabrication being, as expected, associated to polymer and solvent selection and combination of compatible processing methodologies. In general, the applications of scaffolds depend on their mechanical and biological properties and, as such, many possibilities have emerged in recent years.

## 6. Applications of Fiber–Hydrogel Composites 

Recently, research on fiber–hydrogel composites has increased significantly. These scaffolding systems have been investigated for a range of applications, including wound healing [206], regeneration of corneal stroma [184], nucleus pulposus regeneration [201], bone tissue engineering [207], antibiotic delivery [208] and heart valve tissue engineering [209]. Scaffolds with an architecture that mimics native ECM and allows cell infiltration and differentiation has emerged as a prospective solution for the treatment of various health complications. For instance, the fibrous structure in fiber–hydrogel composites is considered of enormous importance for a greater efficiency of the scaffold. This is because tissues have biological fibers with specific composition and architecture that contribute to the normal function of the tissues. Thus, with this, it is possible to simulate biological fibers, approximating the foreign scaffold to living tissue and, thus, enhance cellular growth and maturation (e.g., cell differentiation) [184]. In the following sections, we will contextualize and list in more detail some recent examples of fiber–hydrogel composites applied in wound healing and drug delivery. 

### 6.1. Wound Healing

Wound healing is a complex physiological response that involves a cascade of cells, matrix components and other biological factors [16]. In healthy people, wound healing includes four important phases: hemostasis, inflammation, proliferation, and remodeling. This complex process allows skin functions to be restored. Wounds that fail the normal healing process in a predictable amount of time are considered chronic wounds (CW) [210,211]. Currently, wound care is based on the application of a wide variety of wound dressings (gauzes, absorbent cotton and bandages), debridement, vacuum assisted closure and grafts. Even though they are considered the therapy of choice, wound dressing have some limitations, they are incapable of maintaining the moist environment necessary for wound healing and tend to adhere to the wound, which may cause discomfort to the patient when the dressing is removed [212]. CW treatments are often associated to high economic costs, an increase in surgical procedures and the greater susceptibility of the patient to infection. Microorganisms such as *Acinetobacter baumannii*, *Enterococcus faecalis*, *Pseudomonas aeruginosa* and *Staphylococcus aureus* have the ability to colonize and infect wounds, which complicate the healing process [6,210]. In the most severe cases, patients with infected wounds, such as diabetic foot infections, include mainly antibiotics in their therapy [213]. The impact of excess and inappropriate use of antibiotics has been explored in relation to the various adverse effects, such as bacterial resistance, which has been highlighted as a serious global concern [135]. Several alternatives have been developed for a more efficient wound healing in order to prevent infection to evolve and, in the case of CW, to try and shorten the treatment period [212,214,215,216,217]. There are some properties that ideally a modern wound dressing should have, specifically, the capacity for mechanical protection and adaptation to the shape of the wound, without adhering to wound tissue per se, so as not to cause pain to the patient when removed. Absorption capacity, cytocompatibility, flexibility, ability to ensure a balanced moist environment, induce wound healing, facilitate ECM regeneration, protect the wound from external contaminants and promote debridement are also important features in the development of an effective wound dressing [6,33,166,212]. Wound dressings can be classified based on the affinity of the dressing with the wound into four distinct groups: passive, interactive, advanced and smart dressings [211]. Modern dressings take the most varied forms, including hydrogels, films, sponges, foams, nanofiber mats and, more recently, fiber–hydrogel composites [33,206]. The hydrogel has the ability to absorb exudates and maintain a balance of moisture at the wounded site. In turn, the fiber mimics the fibrous structure of ECM. Since both structures present limitations, the fibers do not facilitate cell migration and hydrogels have low mechanical stability, scaffolds combining both have been the research target of many investigations in order to uncover alternatives for the treatment of wounds [206,217,218]. The combination of the two structure in one scaffold is expected to facilitate healing by generating an environment conducive with cell recognition and attachment (ECM mimicking) with a moist and breathable atmosphere required for a healthy tissue formation. It is known that a large part of mammalian ECM has an aqueous matrix (gel) containing diverse fibrous proteins, essentially collagen, elastin and fibronectin. These proteins surround and guide cells in vivo and act as an anchoring matrix [219,220]. In humans, fibrillar collagen provides tensile strength for ECM, which limits tissue/organ distensibility as is the case of the skin [221]. The ECM is mainly responsible for cell adhesion, migration, proliferation, and regulation of their action. For a complete and effective skin regeneration, it is important that a scaffold is created that mimics the structure and normal skin conditions. Studies have shown that the reinforcement of hydrogels with fibers improves cell function, differentiation and proliferation, as well as structural stability [182,183,195]. Indeed, Schulte et al. described the manufacture of an artificial ECM scaffold consisting of biofunctionalized fibers incorporated in a semi-synthetic hydrogel of HA that allowed the control of cell adhesion [220]. 

There are several polymers used in fiber–hydrogel composites, namely gelatin [206,217,222]. The combination of two separate scaffolds (bilayer scaffold) was studied by Franco et al. for a possible application in skin regeneration. The formulation consisted of a first layer based on a PCL/PLGA membrane (80:20) formed by electrospinning and a second layer of CS/gelatin hydrogel (50:50) crosslinked with glutaraldehyde. The first layer showed excellent mechanical properties and biocompatibility. In the case of the second layer, they obtained a porous structure, capable of swelling more than 500% of its dry size (excellent absorbent properties). The junction of the fibrous membranes provided better mechanical support to the scaffold and, at the same time, reduced the rate of degradation of the layer formed by the hydrogel [222]. In the same light, Zhao et al. through a chemical reaction of the methacrylamide groups with gelatin formed a prepolymer to produce fibers by electrospinning (GelMA). The electrospun GelMA nanofibers were crosslinked by photo-crosslinking, with UV radiation. By manipulating the degree of modification of the gelatin with the methacrylamide groups and the photo-crosslinking time, it is possible to adjust the physical and biological properties. Characteristics such as water vapor permeability, water retention, mechanical resistance and kinetic degradation can be adapted by adjusting the time of UV light radiation. These GelMA scaffolds, which mimic the structure of the native ECM, demonstrated a better orientation of the cellular processes (e.g., cell migration of fibroblasts) and biocompatibility compared to the controls (gelatin and PLGA). The in vivo tests reinforce the potential of this scaffold since it was visible that they accelerated wound repair [217]. Sun et al. went a step further and reported the ability of the GelMA to improve the elastic biodegradable mechanical properties of the construct and its ability to improve cell adhesion, proliferation and vascularization [223]. In turn, Li et al. reports the use of gelatin for the development of a hydrogel fibers. Initially the gelatin-based compound hydrogel fibers were prepared by gel-spinning with PEG6000. Subsequently, the crosslinking agent dialdehyde carboxymethyl cellulose (DCMC) was incorporated in order to improve the thermal and mechanical properties of the hydrogel fibers composed of gelatin-PEG. This scaffold showed a strong capacity to absorb free water due to its 3D structure and porous network. The higher the DCMC content in hydrogel fibers, the more slowly they degrade. In addition, DCMC increased the compatibility of the hydrogel fibers with blood [206]. HA nanofibers are reported to promote wound healing. Due to their high solubility in water, crosslinking is required to increase their water stability. Chen et al. developed an electrospun a mixture of maleicated hyaluronate/poly(vinyl alcohol) methacrylate (MHA/MaPVA) that allowed the formation of mats with the capacity to swell and form fibrous hydrogels. The weight ratio of the nanofiber components influenced the morphology and diameter of the nanofibers. This structure was cytocompatible, promoted cell fixation and displayed high water absorption capacities [218]. PVA has also been combined with PCL to form double layer structures resultant from the combination of PCL nanofibers (hydrophobic) and PVA hydrogel (hydrophilic). After exposure to water, the PVA fiber layer was completely dissolved, and a hydrogel-like structure was formed. Despite this change, the defined shape of the scaffold was maintained due to the stability of the PCL layer in water-based environments. Several aspects were tested in this scaffold, namely, its morphology, wettability, and adhesion and proliferation of mouse fibroblasts. Here, it was seen that fibroblasts exhibited greater proliferative activity on the PCL side of the double layer. In the case of the PVA layer, the same was not seen, which may be a consequence of the greater hydrophilicity of the layer. Based on the behavior and characteristics of the double layer scaffold, the authors concluded that the scaffold had the potential to be used as a dressing or in the prevention of abdominal adhesions [194].

The rapid dissolution of fibers in an aqueous medium becomes a limitation for their application in active wound dressings. In the case of PVP fibers, their rapid solubility remains a problem despite their self-adhesive properties and their ability to incorporate molecules. Recently, to overcome this limitation Contardi et al. proposed to develop PVP-based fiber hydrogels containing hydroxycinnamic acid derivatives. A controlled release of p-cumaric and ferulic acids (derived from hydroxycinnamic acid) from the fibers was observed due to the incorporation of these in the hydrogel. The author also observed in burned skin a reduction in the levels of enzymes known to be positively regulated by reactive oxidative species in burned skin [224]. By electrospinning/electrospraying methods, Azarniya et al. reported the production of a hybrid fiber–hydrogel by combining fibrous mats and hydrogel particles. Through electrospinning, keratin/bacterial cellulose (BC) fibers were produced and simultaneously sprayed with thermosensitive hydrogel particles. The chemically crosslinked hydrogel was composed of non-ionic triblock copolymers (PEO99-PPO65-PEO99; Pluronic F127) conjugated with Tragacanto gum (TG). Due to the low spinning power of keratin, poly(oxide of ethylene) (PEO) was added to the formulation forming the keratin/BC/PEO fibers. Reductions in the diameter of keratin/PEO fibers from 243 ± 57 nm to 150 ± 43 nm and hydrophobicity were observed with the addition of 1% or more of BC. However, despite the reduction of pores, TG and BC modified mats promoted cell fixation and proliferation in fibrous structures. It was seen that the hydrogel particles were uniformly incorporated into the junction of the fibrous network. This modification improved several features of the scaffolds, including hydrophilicity, modulus of elasticity (31%), tensile strength (35%) and ductility (23%) [225]. More recently, Loo et al. developed “intelligent” peptide hydrogels, in which the short aliphatic peptides had the tendency to self-assemble into helical fibers, forming nanofiber hydrogels. These nanofibrous hydrogels were found to possess regenerative properties and to display potential to accelerate the healing of burn wounds [226]. 

### 6.2. Drug Delivery 

In conventional therapies, rapid degradation and excretion of drugs during the circulation process in the body is frequently detected. Consequently, only a small amount of medication will have therapeutic effects in places of interest [227]. Several research groups have focused on the development of new controlled drug delivery systems to allow an effective distribution of drugs in the intended locations at a controlled release rate [193,228]. A drug delivery system is used to transport therapeutic substances in the body more effectively and safely, having the ability to control the amount, the time and the targeted place for drug release [229]. Several scaffolds have been used to encapsulate and deliver therapeutic drugs, namely, fibers and hydrogels [102,230,231,232].

Electrospinning systems allow drugs to be incorporated into the fibers, giving them a high drug loading capacity, increased initial burst, sustained release, and prolonged circulation. Methods of incorporation include blend (or co-, the drug is mixed in the polymer solution), side-by-side (vehicle/polymer solution and the biomolecules are loaded in a separate spinneret), multi-jet (use of multiple nozzles with one or more jets, or a nozzle with different jets), co-axial (two concentric aligned capillaries connected to a high voltage source) and emulsion electrospinning (the drug is encapsulated in an appropriate solvent to be protected from the fiber/solvent system) [48]. Just as there are different ways to incorporate drugs into fibers, drugs may also be released via three distinct mechanisms: desorption of the fiber surface, diffusion in the solid state through the fibers, and fiber degradation [233]. The fiber morphology and its high therapeutic load capacity are beneficial properties that make them potential candidates for drug delivery systems. Electrospun fibers have several advantages especially due to their large surface area and their absorption/release properties [234]. However, large-burst drug release, uncontrolled duration of drug release, and incomplete drug release are recurring problems. The possible agglomeration of bioactive agents on the surface of the fibers becomes a disadvantage of the electrospinning method since it can trigger an initial burst release, which may cause toxicity of the release site [48,224,235]. Such limitations may have implications in the scaffold biomedical goals. 

To incorporate drugs into hydrogels, they can be loaded into the precursor solutions before crosslinking or can be absorbed after gelation [236]. Regarding drug release, swelling is an important property in some stimulus-sensitive drug delivery system. Certain changes in the environment may trigger swelling that allows the release of the drug due to the alterations in mesh size of the polymeric network [237]. Features like hydrophilicity, biocompatibility and tunable mechanical properties are the reason why hydrogels have been used extensively for the controlled release of drugs [193]. Although hydrogels are widely used in controlled release systems, there are some limitations that must be overcome. These scaffolds suffer from low mechanical resistance, which may be responsible for inhomogeneous release [236]. In most hydrogels, their ability to absorb large amounts of water and the presence of large pore sizes may trigger a rapid drug release [208]. In accordance, some investigations have developed/obtained better kinetic release profiles when there is a combination of hydrogels with other structures, namely fibers [193]. The effectiveness of fiber–hydrogel composites for drug administration has been demonstrated [208,227,228]. Nanofiber–hydrogel scaffolds as biofunctionalized platforms appear as attractive alternatives to the ineffective treatments related to direct drug administration.

Persistent neurological dysfunctions are usually triggered by spinal cord injuries due to failure in axon regeneration. Nguyen et al. synthesized lined mats of poly(ε-caprolactone-co-ethyl ethylene phosphate) (PCLEEP) by electrospinning and distributed them in a collagen hydrogel matrix. Both the fibers and the hydrogel contained neurotrophin-3 (model protein) known for promoting neuronal survival, axonal sprouting and regeneration. Additionally, the hydrogel contained miR-222 (model microRNA) known to contribute to the control of local protein synthesis at distal axons. Overtime, it was seen that degradation occurred within the collagen hydrogel, but the PCLEEP fibers maintained their morphology and alignment after 3 months. The composite framework allowed localized and sustained drug/gene delivery, while aligned nanofibers acted to direct remyelination of the injured area. Furthermore, they observed the regeneration of the animal model axon [227]. In a similar study, small fragmented nanofibers of poly(3-caprolactone-_co_-_D_,_L_-lactide) (PCL:DLLA) and collagen were individually dispersed in a hyaluronane-methylcellulose hydrogel (HAMC). These fiber–hydrogel composites were used as a cell-transport system multipotent neural/progenitor stem cells (NSPCs) for the treatment of spinal cord injuries. The results showed that the incorporation of fibers in the HAMC hydrogel influenced the behavior of the NSPC cells, highlighting a better neuronal and oligodendrocytic differentiation in the scaffold PCL:DLLA/HAMC compared to collagen/HAMC [199]. In both studies, the complex generated from the combination of fibers and hydrogels allowed for a faster cell development and consequent regeneration.

A laminated fiber–hydrogel composite based on PCL electrospun fiber mats coupled with poly(ethylene glycol)-poly(ε-caprolactone) diacrylate (PEGPCL) hydrogels processed by UV polymerization was developed to control the release of a model hydrophilic protein (e.g., bovine albumin serum, BSA). To study the release of the hydrophilic protein, BSA was added to the system before crosslinking. The results reported by Han et al. suggested the relevant role of PLC fibers (diameter of approximately 0.45 μm) in the release of the drug in a uniform and delayed manner, by reducing swelling of hydrogels and water penetration rates and by increasing the length of the diffusion path and the diffusivity of the drug. In addition, the bioactivity of proteins after release was proven since extension of PC12 cell neuritis was detected. In general, the PCL fibers in the PEGPCL hydrogel demonstrated an important role in three main areas: control of the release kinetics of the hydrophilic protein, reduction of burst release (initial) and increased duration of drug release (more than two months) [193].

Osteomyelitis is a bone disease caused mainly by methicillin-resistant *Staphylococcus aureus* (MRSA). Various antibiotics are administered to reduce this infection, namely the glycopeptide vancomycin hydrochloride (vanco-HCl). The bacterial plaque that forms around the infected area limits treatment by preventing the diffusion of the antibiotic vanco-HCL to the infected site, which then requires the administration of high doses. This overuse of antibiotics in addition to their impropriate function can lead to systemic toxicity. To try and solve this problem, Ahadi et al. developed a scaffold made of poly(L-lactide) (PLLA) fibers produced by electrospinning followed by aminolysed, encased in a hydrogel of silk fibroin/oxidized pectin. PLLA fibers were loaded with vanco-HCl to promote a more sustainable release of the antibiotic at the affected site, resulting in a 61% reduction in drug release. This scaffold revealed better mechanical properties compared to the single hydrogel (without fibers), namely, a higher crosslinking density (52%), a higher compression module (30%) and a lower expansion rate (15%). Biologically, the fiber–hydrogel composite was seen to have activity against MRSA and to be cytocompatible with cells, largely due to the presence of fibers aminolized with drugs [208]. Ekaputra et al. developed by electrospinning/electrospraying a hybrid mesh of PCL/collagen and HA hydrogel, Heprasil^TM^, loaded with vascular endothelial growth factors (VEGF) and platelet-derived growth factors (PDGF). It was seen that the fiber–hydrogel composite PCL/collagen-Heprasil was successful in allowing a double simultaneous loading of the growth factors VEGF165 and PDGF-BB and to promote their controlled release over a period of five weeks, in vitro [192]. Recently, biocompatible vehicles for the release of the crystal violet drug (CV) have also been described, in which polydopamine microfibers (PDA) were incorporated in a pullulan (PHG) hydrogel crosslinked by poly(ethylene glycol) diglicidyl ether (chemical crosslinker). PDA fibers attributed the pH-responsive drug release behavior to the PHG hydrogel. This happens in response to the acidic conditions, which increase the electrostatic repulsion force between the PDA (protonated and positively charged) and the drug CV (positive charge). This repulsion promotes the release of the drug, with a detectable a cumulative release of 60.3% (pH 7.4), which increased to 87% with a decrease in pH to 5. In addition, the incorporation of PDA fibers and the adjustment of their content allowed to regulate several properties of the composite PHG-PDAs, namely, its viscoelastic characteristics, mechanical performance, mesh size and swelling/disintegration properties of the PHG hydrogel. The developed scaffold proved to have great potential to be used in drug delivery systems, given its good cytocompatibility, non-toxicity and easily adjustable properties for a controlled release of CV [238]. Overall, data demonstrated the ability of the engineered systems to promote a controlled drug delivery, in which the fibrous mesh guaranteed the mechanical stability of the construct while the hydrogel released the loaded active compounds.

A new physical approach based on hydrogel and nanofibers (or NEEDs) for cell encapsulation has been described in the work of An et al. Here, tubular constructs with different compartments were developed, consisting of Nylon 6,6 nanofibers, manufactured by electrospinning, being subsequently impregnated in different hydrogel precursor solutions (alginate, chitosan or collagen) and crosslinked. Fibers had an average diameter of 200 nm with 1 μm interconnected pores. Compartmentation proved to be an asset for co-encapsulation, co-culture and co-distribution of different individual cells and cellular aggregates (islets), with cell viability being observed. Finally, the potential application of NEEDs for cell therapies using a type 1 diabetic model was tested, and the disease was corrected (in 8 weeks), which proved the therapeutic potential of NEEDs within primary rat islets (without the disease) [228].

In wound healing, it is important to pay attention to the biomaterials used to produce wound dressing. To achieve the desired objectives, the properties of each biomaterial are optimally combined. Studies have shown that local administration of therapeutic agents through wound dressings can improve the wound healing process [16]. In fact, a bioactive dressing of fibers of silk fibroin (SF) produced via electrospinning was developed and then combined with the alginate hydrogel (ALG) capable of supplying amniotic fluid (AF). This dressing had the ability to release AF, highly enriched with various therapeutic agents, at the wound site. The AF release profile was related to the concentration of ALG (greater release of AF in lower amounts of ALG). The increase in cell proliferation and collagen dissemination and secretion due to AF in fibroblast cultures strengthens the potential of the SF/ALG fiber–hydrogel composite to accelerate the healing process in severe wounds [239]. In a similar study, a bi-layer dressing of gelatin nanofiber mats loaded with epigallocatechin gallate (EGCG)/PVA hydrogel was produced for the treatment of acute wounds. The hydrogel was used as a protective and hydrating outer layer of the bi-layer dressing. Jaiswal el al. observed that the decrease in crosslinking time led to a slower EGCG release profile. This increased in the 2-4 days release period demonstrating the ability of this scaffold to guarantee a gradual drug release. Faster wound contraction, improvement in angiogenesis, reepithelization and less inflammatory response compared to control were also observed [240]. More recently, Chen et al. developed CS/gelatin hydrogels with polydopamine-intercalated silicate nanoflakes (PDA-Silicate). These were electrospun in the form of nanofibers loaded with the antibiotic tetracycline hydrochloride (TH). In this sandwich-like nanofiber/hydrogel composite (NF-HG) the incorporation of the fibers in the hydrogel resulted in a restriction in the release of antibiotic TH. However, it allowed a sustained release rate of TH in NF-HG for long-term protection. In addition, this structure reduced the toxicity of the drug associated to the rapid release. Furthermore, the excellent adhesiveness and anti-infectious properties demonstrated by the NF-HG, turned this formulation particularly attractive to be used as a wound dressing [241].

## 7. Conclusions

The world of biomaterials, specifically polymers, continues to significantly impact on the field of biomedicine. The diversity of polymers and the different ways of using them in scaffolds have evolved considerably in the last years, proposing active solutions for daily problems. In recent decades, combinations of different scaffolding systems in one solution have been researched, demonstrating great potential in wound healing and drug delivery systems, particularly in the fight against antibiotic-resistant pathogens. Indeed, hydrogel and fiber composites have been engineered as effective therapies, overcoming many of the mechanical, physical, and biological limitations of fibers and hydrogels when used in individual systems. Although research in this field is still very limited and is basically taking the first steps, the potential is clear. In the next years, it is expected the research on these composites to continue evolving and growing, as the need for more adaptable and specialized biomedical devices grows as well.

## Figures and Tables

**Table 1:** Origins and main properties of natural and synthetic polymers commonly used in wound healing, tissue engineering and drug delivery applications.

Polymer		Structural Formula	Origin/Synthesis Pathway	Main Characteristics	Known/Key/Main/Selected Applications	Reference
**Natural**	**Hyaluronic acid**	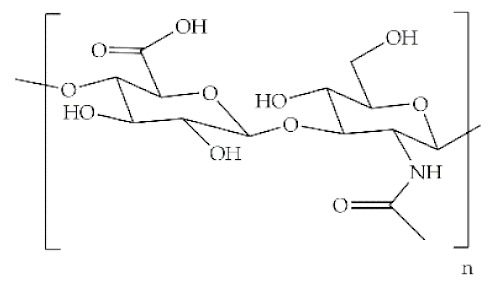	Connective tissues of any vertebrate	Non-sulfated anionic glycosaminoglycan; linear conformation; hydrophilic; water-soluble; highly viscoelastic; non-immunogenic; biodegradable	Wound healing; biomolecule (e.g., ocatdecyl acrylate) delivery; cartilage/bone regeneration; bioink in 3D printing	[11,21,22,23,24]
	**Chitosan**	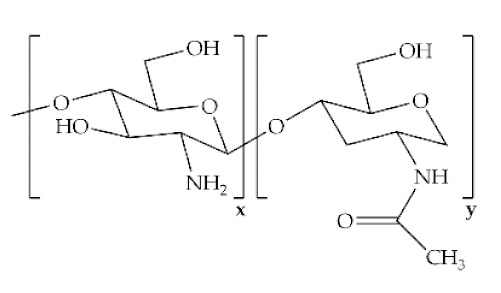	Chitin (mostly found in the exoskeleton of shrimps, crabs, lobster and squid pens; cuticles of insects; and in lesser amounts, in cell walls of fungi, yeast and plants)	Cationic linear polysaccharide; hydrophilic; pH-dependent charge density; physicochemical properties dependent on the degree of acetylation, crystallinity, molecular weight and degradation; non-toxic; biodegradable; non-antigenic; biologically adhesive; hemostatic effect; antimicrobial; anti-inflammatory	Wound healing; bone/cartilage regeneration; antibiotic/antibacterial agents/growth factors delivery	[20,22,25,26,27]	
	**Alginate**	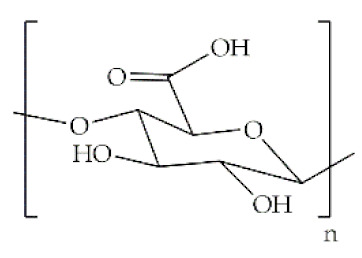	Brown seaweed or bacteria (*Azotobacter* and *Pseudomonas* specie)	Anionic linear polysaccharide; slow gelation time; hydrophilic; water soluble; low toxicity; low cost; water retaining capacity; biodegradable	Wound dressings; burn treatments; protein/small chemical drug delivery; bone/cartilage regeneration	[5,28,29,30]
	**Cellulose**	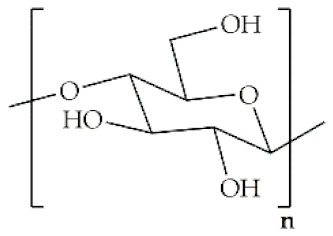	Plants (mainly derived from cotton fiber, dried hemp and wood), bacteria (e.g., *Acetobacter*, *Azotobacter*, *Rhizobium*, *Agrobacterium*, *Pseudomonas*, *Salmonella*, *Alcaligenes* and *Sarcina ventriculi* species)	Linear homopolysaccharide; hydrophilic; rigid; fibrous morphology; relatively easy extraction; non-toxicity; low cost; biodegradable	Bone/tendon tissue regeneration; wound healing; loading antimicrobial agents and antibiotics	[31,32,33,34]
	**Gelatin**	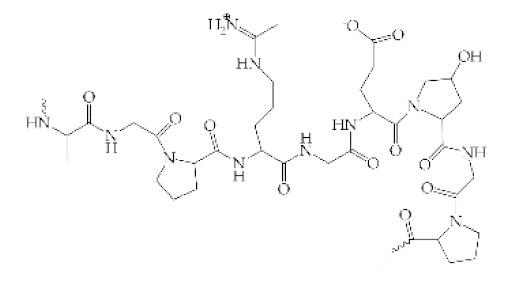	Skin and bone of bovine and porcine, fish and marine organisms (incomplete denaturalization of collagen)	Linear polypeptide; hydrophilic; water soluble (35 °C); soluble in polyhydric alcohols and several other organic solvents; cost efficient; easily available; biodegradable; non-antigenic; similarity to collagen	Wound healing; bone regeneration; articular cartilage repair; tendon tissue engineering	[26,35,36,37]
	**Collagen**	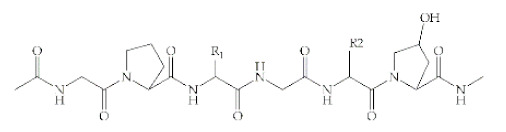 Collagen type I	Animals (e.g., Achilles tendon, bovine skin, porcine skin, and human cadaveric skin)	Polypeptide; good surface-active agent; enhanced water holding capacity; highly hydrophilic; twenty-eight different collagen types; low antigenic and cytotoxic responses; antioxidant; biodegradable; most abundant protein of animal origin	Wound healing; tissue replacement and regeneration (bone, cartilage, skin, blood vessels, trachea, esophagus); carriers for drug/protein delivery	[38,39,40,41]
**Synthetic**	**Poly(ethylene oxide)**	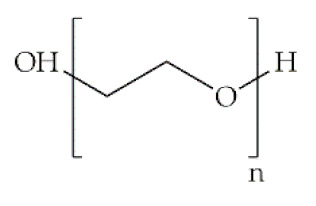	Anionic ring-opening polymerization of ethylene oxide (EO)	Neutral polymer; hydrophilic; water soluble; low toxic; biodegradable	Gene/drug delivery systems; biomedical implants; neocartilage tissue formation; transdermal delivery	[42,43,44,45]
	**Poly(ε-caprolactone)**	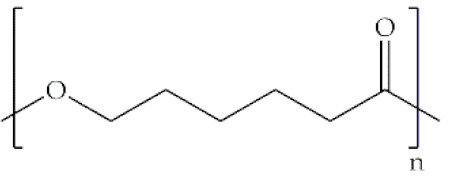	Ring-opening polymerization of ε-caprolactone monomer using a wide range of catalysts	Semicrystalline; hydrophobic; excellent mechanical strength; slow degradation rate; nontoxic; biodegradable	Tendon tissue engineering; skin regeneration; vascular scaffolds	[37,44,46,47]
	**Polylactic acid**	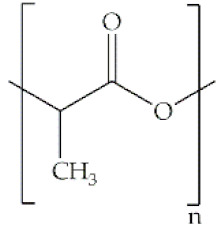	Polycondensation of lactic acid and ring opening polymerization of cyclic lactide	Thermoplastic aliphatic polyester; hydrophobic; poor ductility; low strength; bioabsorbable; biodegradable	Ligament and tendon repair; vascular stents; bone regeneration	[5,48,49,50]
	**Poly(lactic-co-glycolic acid)**	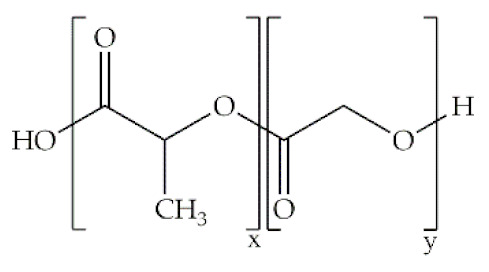	Ring-opening polymerization of lactide	Linear aliphatic copolymer; relatively hydrophobic; enhanced flexibility; thermal processibility; tunable degradation/biodegradation; minimal side effects	Wound healing; bone/ cardiac/periodontal tissue regeneration; protein/growth facto/antibiotic/ gene delivery	[51,52]
	**Poly(vinylpyrrolidone)**	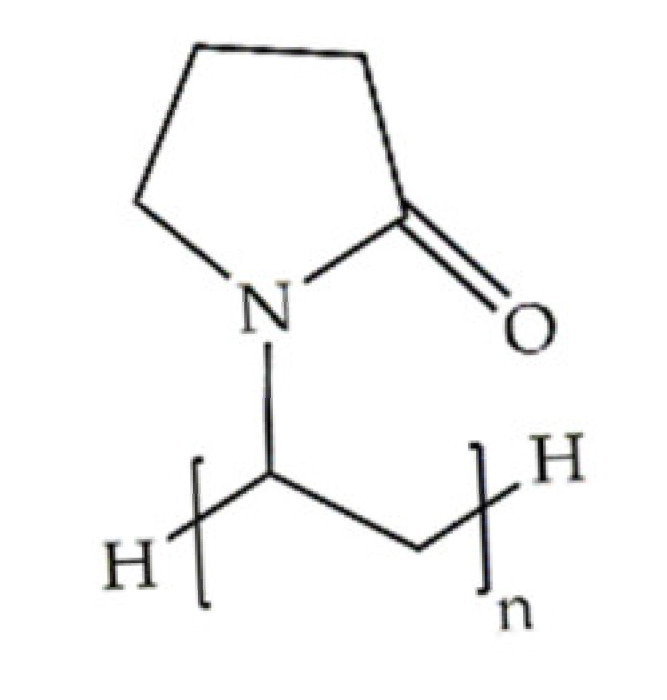	Free radical polymerization from the vinylpyrrolidone monomer	Neutral polymer; amorphous; hydrophilic; water soluble; stable; nontoxic; adhesive power; non-biodegradable	Wound healing; gene delivery; biomedical implants (orthopedic, dental, vaginal, breast); neural/cardiac/pancreatic tissue regeneration	[17,53,54,55]
	**Poly(vinyl alcohol)**	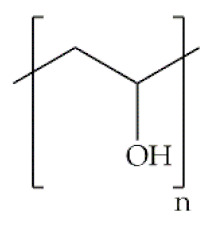	Vinyl acetate with base catalyzed transesterification with ethanol	Linear polymer; hydrophilic; semicrystalline; water soluble; pH sensitive; high swelling capability; excellent chemo-thermal stability; transparency; high tensile; strength; high elongation at break; flexibility; non-toxic; non-carcinogenic and bioadhesive properties; non-biodegradable	Drugs/protein/growth factor/nanoparticle/gene delivery; skin healing and reconstruction; kidney regeneration	[48,56,57]

**Table 2:** General classification of hydrogels considering their source, polymers charge, polymer composition, structural configuration, degradation, physical properties, response to stimuli, and type of crosslinking [68].

Hydrogels Classification	
**Source**	Natural, synthetic or hybrid
**Charge of polymers**	Ionic, non-ionic, amphoteric or zwitterionic
**Polymeric composition**	Homopolymer, copolymer, multipolymer, IPN or semi-IPN
**Configuration**	Amorphous, crystalline or semicrystalline
**Degradability**	Biodegradable or non-biodegradable
**Physical properties**	Conventional or smart
**Response**	Physical, chemical, or biochemical/biological
**Type of crosslinking**	Chemical or physical

**Table 3:** Properties and limitations of different types of physical and chemical crosslinking.

Hydrogels	Crosslinking Engine	Concept	Advantages	Disadvantages	Reference
**Physical**	**Ionic/Electrostatic Interaction**	Interaction between a polyanion and a multivalent cation or a polycation, and vice versa (interaction between opposite charges)	Simple method; self-healing ability	Low stability in physiological environments and limited mechanical strength	[29,94,97,98]
	**Hydrogen** **Bonding**	Hydrogen bond between polymer chains (electron-deficient hydrogen atom and a high electronegativity functional group)	Absence of chemical crosslinkers	High dilution and dispersion rate over a few hours in vivo	[85,99]
	**Hydrophobic** **Interaction**	Polymers with hydrophobic domains are capable of crosslinking in aqueous environments by means of reverse thermal gelation (“sol-gel”) (increased temperature leads to the aggregation of these domains)	Shape memory; autonomously self-healing properties; high degree of toughness	Poor mechanical properties	[99,100,101]
	**Crystallization**	The principle of freezing polymers at low temperatures, followed by thawing at room temperature causes the formation of crystals which leads to the formation of hydrogels	Stability and mechanical properties can be increased with increasing the freezing time and freeze–thaw cycles; simple method; not require additional chemicals and high temperature	Freeze/thaw processes applied for long periods of time can alter the behavior of the hydrogel	[96,102,103,104]
**Chemical**	**Photo-crosslinked**	The crosslinking of monomers or oligomers is initiated in the presence of an irradiation of UV/visible light and a photoinitiator that, when absorbing photons, is cleaved and forms free radicals that trigger polymerization	No toxic crosslinking agents are required; excellent spatial and temporal selectivity; low processing cost and energy requirements	The photoinitiator can produce free radicals with effects on immunogenicity and cytotoxicity responses	[105,106]
	**Enzymatic** **Reaction**	Certain enzymes (e.g., transglutaminases, horseradish peroxidase and tyrosinase) help to catalyze crosslinked reactions between two or more polymers	Mildness of the enzymatic reactions at normal physiological conditions; high efficiency; selectivity; non-toxicity; good biocompatibility; fast gelation process; tunable mechanical properties	Instability and poor availability of some of the enzymes	[107,108,109]
	**Crosslinking** **Molecules**	Crosslinkers (e.g., glutaraldehyde, carbodiimide agents, genipin and citric acid) are small molecules with two or more reactive functional groups responsible for the formation of bridges between polymers chains	Easiness and versatility method	Possible cytotoxicity of the crosslinking agent (e.g., glutaraldehyde)	[110,111,112]
	**Polymer-Polymer**	Crosslinking reaction occurs between pre-functionalized polymer chains with reactive functional groups under favorable conditions. Polymer–polymer bonds can be formed by Schiff bases and by Michael addition reactions	Not using crosslinking molecules	Requires the modification of the polymer chains before their conjugation	[113,114]

**Table 4:** Examples of hydrogel crosslinking systems employed in wound dressing, tissue engineering and drug delivery.

Hydrogels	Crosslinking Engine	Hydrogel Composition	Applications	Reference
**Physical**	Ionic Interaction	6-PG-Na^+^-crosslinked CS	Drug delivery; wound dressing	[115]
		CaCl_2_-crosslinked alginate-pectin	Wound dressing	[30]
		Poloxamer-heparin/gellan gum	Bone marrow stem cells delivery	[117]
		Al_3_^+^-crosslinked cellulose	Drug delivery	[118]
	Hydrogen Bonding	PVA/poly(acrylic acid)	Surgical sutures and load-bearing fields	[119]
		1,6-hexamethylenediamine (HMDA)-crosslinked cytosine and guanosine modified HA	Injectable drug delivery; soft tissue engineering; regenerative medicine	[120]
	Crystallization	PVA/poly(ethylene glycol)	Wound dressing	[121]
		CS/PVA	Anti-inflammatory drug loading and release	[102]
		PVA/cellulose	2-layered skin model	[122]
**Chemical**	Photo-crosslinked	PEGDA	Tissue engineered heart valves	[123]
		GelMA	Tissue engineering; drug delivery; regenerative medicine	[124]
		GelMA/PEGDA	Bone regeneration	[116]
		GelMA/CS	Tissue engineering	[60]
	Enzymatic Reaction	Horseradish peroxidase -crosslinked HA/silk fibroin	Tissue engineering	[21]
		Horseradish peroxidase -crosslinked Silk fibroin- tyramine-substituted silk fibroin or gelatin	Cell delivery	[125]
		Transglutaminase-crosslinked gelatin–laminin	Neuromuscular tissue engineering	[126]
	Crosslinking Molecules	Genipin-crosslinked CS	Drug delivery systems in oral administration applications	[111]
		Genipin-crosslinked CS/gelatin	Drug delivery	[127]
		Glutaraldehyde-crosslinked CS	Tissue engineering	[128]
	Polymer–Polymer	CS/Alginate	Neuronal tissue engineering	[129]
**Hybrid**	Chemical Crosslinking followed by Crystallization	Ethylene glycol diglycidyl ether-crosslinked microcrystalline Cellulose/PVA	Drug delivery	[130]

**Table 5:** Examples of natural and synthetic fibers [135,153].

Type of Fibers		
**Natural**	**Plant**	Bast fibers (e.g., jute and flax); seed fibers (e.g., cotton and coir); leaf fibers (e.g., banana and abaca); grass fibers (e.g., sugarcane bagasse and bamboo); straw fibers (e.g., rice, corn and wheat); wood fibers (e.g., softwood and hardwood)
	**Animal-Based**	Wool; silk; hair
**Synthetic**	**Inorganic**	Metals and alloys (e.g., metals fiber); metal or semi-metal compounds (e.g., glass and ceramics fibers); carbon-based fibers (e.g., carbon and graphene fibers)
	**Organic**	Synthetic polymers (e.g., polyamide nylon, polyethylene terephthalate, phenol-formaldehyde, PVA, polycarbonate, polyvinyl chloride and polyolefins (polypropylene and polyethylene)); natural polymer (e.g., chitosan and alginate)

**Table 6:** Set up, concept, advantages and disadvantages of the most common fiber manufacturing techniques, namely, electrospinning, wet-spinning, melt-spinning and dry-spinning.

	Electrospinning	Wet-Spinning	Melt-Spinning	Dry-Spinning
**Set Up**	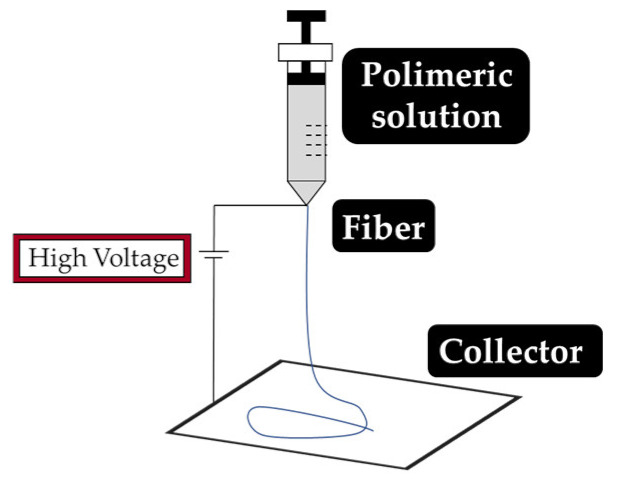	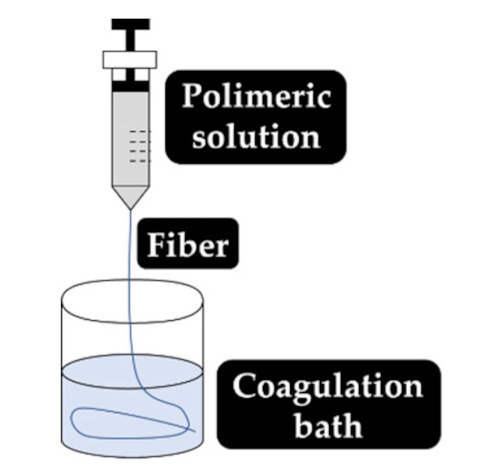	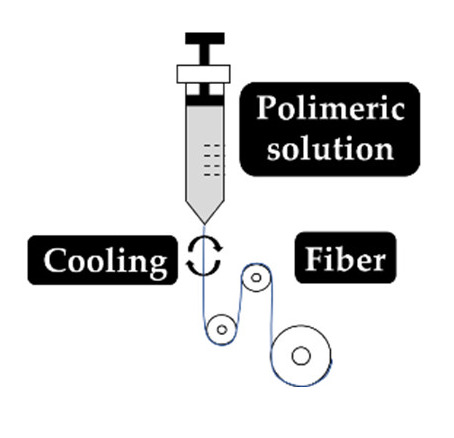	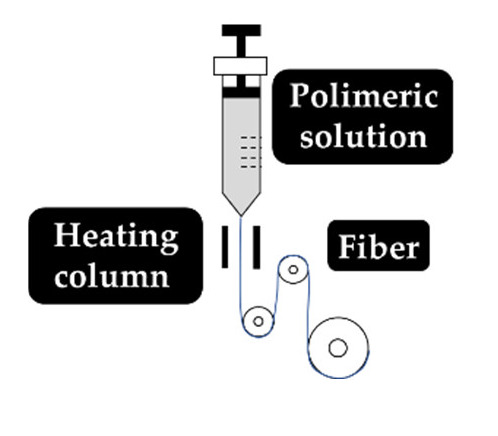
**Concept**	The polymer dissolved in an appropriate solvent is injected by a needle towards a collection plate. Due to the high applied electric field, potential difference generated between the syringe (acts as an electrode) and the plate (acts as an electrode count), the polymer is attracted by the collecting plate, and the polymer solution is converted into nanofibers	The polymer is dissolved in an appropriate solvent and later injected through a fiery into a coagulation bath containing a non-solvent liquid. In the coagulation bath, continuous polymerization of the filaments occurs. After the formation of the fibers, they are extracted from the coagulation bath by means of rollers-induced capture	The solid polymer is heated above its melting point within the extruder and is then expelled through a die, solidifying on cooling. In a pick-up, the fibers are then recovered and mechanically stretched	The polymer is dissolved in a suitable solvent (must be highly volatile). The initial solution is injected through the spinneret and through a heating column that causes the solvent to evaporate. Consequently, the polymer solidifies, and dry fibers are attained
**Advantages**	Fibers with a large surface area, high porosity, great flexibility, and excellent mechanical properties; simple and straightforward process; cost efficiency	Wet-spun structures have greater intrinsic porosity and larger interconnected pores; versatile technique in terms of material selection	Fabrication process is quick; Not require added solvents	Enables spinning of polymers vulnerable to thermal degradation
**Disadvantages**	Fiber thickness increases density and reduces pore size in 3D structures that can limited the interaction of cells with the fibers; toxicity of the solvents and the instability of the jets; slow process	Long exposure to chemicals during the processing and coagulation may impact negatively on the cells’ microenvironments	Limited to thermically-resistant polymers; unstable in the production of fine fibers	Requires high temperatures which can affect the properties/characteristics of the fibers/fiber surface
**Ref.**	[164,165,166,167,168,169,170]	[171,172,173,174]	[175]	[176,177]

## Data Availability

Not available.
